# Dynamics of Dynamics within a Single Data Acquisition Session: Variation in Neocortical Alpha Oscillations in Human MEG

**DOI:** 10.1371/journal.pone.0024941

**Published:** 2011-09-22

**Authors:** Qian Wan, Catherine Kerr, Dominique Pritchett, Matti Hämäläinen, Christopher Moore, Stephanie Jones

**Affiliations:** 1 McGovern Institute for Brain Research, Massachusetts Institute of Technology (MIT), Cambridge, Massachusetts, United States of America; 2 Harvard Osher Research Center, Harvard Medical School, Boston, Massachusetts, United States of America; 3 Athinoula A. Martinos Center for Biomedical Imaging, Massachusetts General Hospital, Charlestown, Massachusetts, United States of America; City of Hope National Medical Center and Beckman Research Institute, United States of America

## Abstract

**Background:**

Behavioral paradigms applied during human recordings in electro- and magneto- encephalography (EEG and MEG) typically require 1–2 hours of data collection. Over this time scale, the natural fluctuations in brain state or rapid learning effects could impact measured signals, but are seldom analyzed.

**Methods and Findings:**

We investigated within-session dynamics of neocortical alpha (7–14 Hz) rhythms and their allocation with cued-attention using MEG recorded from primary somatosensory neocortex (SI) in humans. We found that there were significant and systematic changes across a single ∼1 hour recording session in several dimensions, including increased alpha power, increased differentiation in attention-induced alpha allocation, increased distinction in immediate time-locked post-cue evoked responses in SI to different visual cues, and enhanced power in the immediate cue-locked alpha band frequency response. Further, comparison of two commonly used baseline methods showed that conclusions on the evolution of alpha dynamics across a session were dependent on the normalization method used.

**Conclusions:**

These findings are important not only as they relate to studies of oscillations in SI, they also provide a robust example of the type of dynamic changes in brain measures within a single session that are overlooked in most human brain imaging/recording studies.

## Introduction

Oscillatory dynamics in neocortex are thought to be important correlates of neurological states such as arousal [1], attention [2], [3], [4], [5], [6], sensory perception [7], [8], [9], [10], memory processes [11], [12], and readiness to learn [13]. They are also disrupted in neurological disorders [14], [15], [16]. The mechanistic underpinnings of such oscillations [2], [17], [18], [19], and their meaning for perception [20], are a current topic of intensive debate in systems neuroscience.

Electroencephalography (EEG) and magnetoencephalography (MEG) provide high temporal resolution and noninvasive means of measuring oscillatory dynamics in humans. In recent studies, we have used MEG to investigate two components of the ‘mu’ rhythm in primary somatosensory neocortex (SI), mu-alpha (7–14 Hz) and mu-beta (15–29 Hz). We have examined the natural expression patterns of these oscillations, explored their potential detailed mechanistic underpinnings [8], and observed systematic alterations in oscillations strength in aging [21]. We have found that the expression of these oscillations is correlated with perceptual detection of threshold-level tactile stimuli [2], [8]. Further, we observed attention-induced modulation of the SI mu rhythm dominated by post-cue allocation of the alpha component [2], which can be enhanced with perceptual learning associated with meditation practice [22].

Significant variation in the expression of such oscillatory neocortical dynamics has been known to exist on the time scale of hours: Implicit and explicit learning paradigms can shift the expression pattern of oscillations on this time scale [23], [24], [25], and individual subjects cycle through epochs of relative vigilance and arousal [1], [26]. Yet changes in these dynamics over the course of an hour-long experimental session are rarely explicitly considered in experimental design and data analysis. These shorter-term changes are usually treated as noise and dealt with by taking averages over the entire session (e.g., of event-related signals). However, such dynamics may impact the inferences made from studies by introducing a latent session-duration dependent variable that lowers statistical power, and obscures subtleties that only emerge at certain epochs within an experiment.

As an object case in the potential impact of such within-session dynamics on human studies, here we describe within-session dynamics in the expression of alpha oscillations in SI during a cued-attention task. This investigation was motivated by our prior observation that aspects of neocortical dynamics were most robust toward the end of the experiment [2], [8], [27]. In the current analysis, we found that absolute alpha power and its modulation driven by selective somatotopic attention cueing, both evolved within a session. Our results suggested that short-term learning effects that alter cortical rhythms take place even over a single hour of data collection in a paradigm that has no explicit learning requirement. Further, we describe how different approaches to baseline normalization can impact these observations, particularly at the time of the cue. These methods and data have bearing on the analysis and use of MEG as a tool for investigating oscillation dynamics, as well as any human studies—using fMRI, EEG, MEG or other modalities—that require averaging across sessions greater than an hour in length.

## Methods

The data reported in this paper were collected for a prior study, and detailed methods for experimental paradigm and data acquisition can be found in Jones et al. (2010) and Kerr et al. (2011) [2], [22]. Here, we review these prior methods and provide details on current data analysis techniques. We restricted our analysis to the alpha frequency band as our prior research revealed that attention had a more significant effect on alpha than beta frequencies [2], [22].

### Subjects

Twelve adults, one male and eleven females, between 18–50 years old (mean age  = 31.6 years, S.D.  = 7 years), participated in the study. Selection criteria included being neurologically healthy (excluding any musculoskeletal diseases, arthritis, lupus, multiple sclerosis, scleroderma, and diagnosed current psychiatric disorder), right-handed, medication free or on stable doses of SSRI medication. The experimental protocol was approved by the Internal Review Boards of the Massachusetts General Hospital and Harvard Medical School. All subjects gave signed informed consent agreeing to participate in the study.

### Stimuli

Subjects' left hands and left feet were rested on solid plastic frames throughout the experiment. A fused multi-layer piezoelectric bender was built into the frame of each stimulator that delivered the stimuli (single cycle of a 100 Hz sine wave, 10 ms duration) to the distal pads of the 3^rd^ digit of the left hand or 1^st^ digit of the left foot, via a delrin contractor affixed to the piezoelectric (7 mm diameter presented within a 1 cm circular rigid surround). The devices were not attached to the skin. Stimulus strength was dynamically manipulated using a Parameter Estimation Sequential Testing (PEST) convergence procedure as described in detail in [8], [28], [29], which was designed to maintain the stimulation strength at a set detection threshold as discussed below.

### Experimental Procedure

#### Localization Runs

In the current study, we report only on activity from the hand area of SI contralateral to the side of hand stimulation, as in Jones, et al. (2010) [2]. To localize a primary equivalent current dipole (ECD) in the hand area of contralateral SI, each experimental session began with a run of 60 trials of suprathreshold stimuli (100% detection rate) delivered to the 3^rd^ digit of the left hand (2 minutes of stimulus with an ISI of 3 seconds).

#### Cued Attention Runs

**Figure 1:**
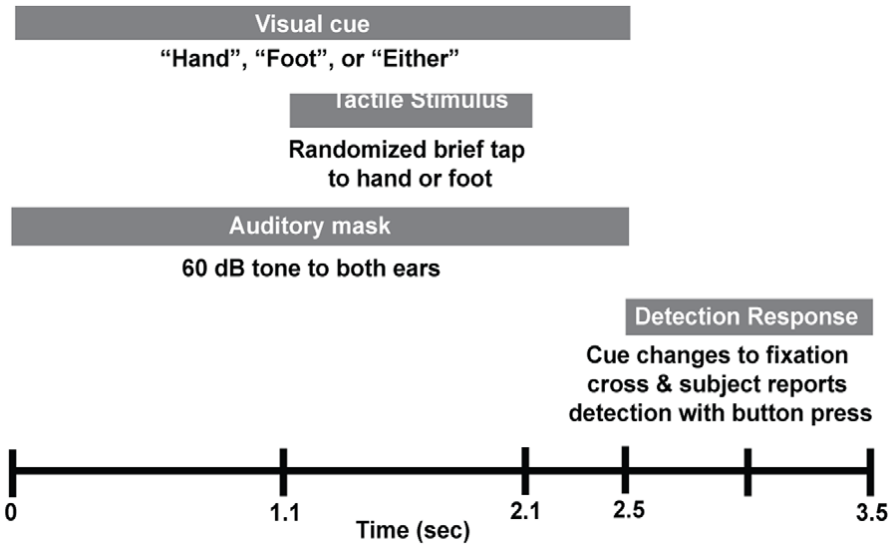
Experimental Design for Cued Detection Runs. Reproduced with permission from Jones et al 2010 [2].

Figure 1 illustrates the experimental paradigm. Subjects were instructed to fixate straight ahead on a cross on a projection screen. A trial began when the cross turned into a word, directing the subject to attend to the ‘Hand’ (attend-hand condition), the ‘Foot’ (attend-foot condition), or ‘Either’ location. At a randomized time between 1.1 s to 2.1 s after the onset of the visual cue (at fixed 100 ms intervals), the piezoelectric stimulator delivered a tactile stimulus to either the finger or the toe or neither location. The stimulus presentation was balanced between finger and toe and the order the stimuli were presented was randomized with an event related design so that the subject could not predict the sequence or timing of stimuli. The visual cue onset was also accompanied by a 60 dB, 2 kHz tone delivered to both ears to mask audible noise created by the piezoelectric tactile stimulators. The auditory and visual cues continued for 2.5 s. At the end of the 2.5 s visual cue (and the auditory tone), which was at least 400 ms after the stimulation, subjects were instructed to report detection or non-detection of the stimulus at the cued location, using a button press with the second and third digits of the right hand respectively. The trial ended 1 s after the termination of the visual/auditory cue, at which time the next cue was presented to start the next trial.

The strength of the stimulation was maintained with a PEST procedure fixed at a detection threshold stimulus rate of 66% throughout the cued attention runs for both the finger and toes stimulation. In addition, 10% supra-threshold (100% detection rate) stimuli and 20% null-stimuli were randomly interleaved into each cued-attention run for each stimulus condition.

There were 120 trials per run, 40 of each attention condition, randomized in presentation order. Subjects were given a short practice run before the start of recording. Each subject underwent an average of 7 (S.D.  = 1 run) runs with small breaks in between each run, accumulating an average of 774 (S.D.  = 83 trials) total trials, 269 (S.D.  = 35 trials) trials in each condition after artifact rejection, with the experimental session lasting approximately one hour (t = 50 minutes, S.D.  = 5 minutes).

#### MEG Data Acquisition

The MEG data were acquired using a 306-channel whole-head planar dc-SQUID Neuromag Vectorview system (Helsinki, Finland) and a sampling rate of 601 Hz. Data were filtered from 0.1 to 200 Hz. Four coils recorded head position for co-registration with structural MR images. Vertical and horizontal electro-oculogram (EOG) signals were recorded for eye-movement artifact rejection. Thresholds for prominent EOG and stimulus artifact rejection were set by manual inspection. This is slightly different than in Jones et al. (2010), where the EOG threshold was fixed at 100 µV [2]. This difference did not significantly change the results. Only trials with EOG artifacts that were found in the period of interest ([0, 1100] ms post-cue) were discarded. Most subjects complied with instruction to blink during the response period, which is not included in the period of interest, therefore very few trials were rejected due to EOG artifacts due to blinks.

#### Source Analysis

The source analysis was automated by Xfit, a standard commercial software within the bundle of the Elekta Neuromag Software Suite (product of Elekta Neuromag Oy, Helsinki, Finland). Xfit was used to estimate an equivalent current dipole (ECD) source in SI at the time of peak activity calculated as the maximum response occurring at <100 ms post-stimulus (mean peak activity  = 66.8 ms, S.D.  = 6.4 ms) in the mean signal from hand localization runs described above (suprathreshold stimuli with minimum of n = 50 trials for each subject). The goodness of fit of the forward solution from a single SI localized ECD to the recorded data was larger than 70% in all fit data at the time of the peak response. The location of the SI source was co-registered with the individual's anatomical MRIs and it was confirmed that the source generated by the hand stimulation emerged from the anterior bank of the post-central gyrus finger representation of area 3b in SI [30] in all subjects. See for [2], [8], [27] example localizations. As in our prior studies [2], [8], [21], [22], [27], all of the analyses presented here used the forward solution from this localized SI source.

A customized MATLAB script was written to extract broadband signal activity from the forward solution of the estimated SI ECD, as well as signals used for EOG rejection and alignment of trials across triggers.

### Data Analysis

#### Calculation of Spectral Power

A complex wavelet analysis was calculated using a complex wavelet algorithm that determined near instantaneous changes in time-frequency representations (TFRs). The TFRs were calculated from 1–40 Hz on the SI ECD time courses by convolving signals with a complex Morlet wavelet of the form 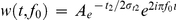 for each frequency of interest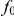, with 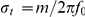, and *i* the imaginary unit. The normalization factor was 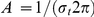, and the constant *m* defined to be 7, thus allowing a compromise between time and frequency resolution, as seen in [8]. Time-frequency representations (TFRs) of the power were calculated as the squared magnitude of the complex wavelet-transformed data averaged from [−667, 2333] ms around cue for each trial. In all analyses, alpha power was calculated by averaging across the 7–14 Hz band from the TFR.

### Methods to Quantify Changes in Brain Activity Across the Experiment

#### Alpha Power Changes

For each subject, total alpha power was calculated by first averaging across the time period of [−667, 2333] ms around cue for each trial, and then all trials were grouped into bins of 50 chronologically. Due to the fact that the length of experiment varied by subject, trials that did not fit into bins of 50 were disregarded in the analysis. To facilitate comparison across subjects, the averages of each bin were normalized to the first bin for each subject. A regression analysis was performed between power and bin number across the experimental session, and the data was fit to a linear, a quadratic, and an exponential model to examine the evolution of power over time. In addition, to be consistent with our later analyses, the entire experimental session was also divided into three blocks: Early (E), middle (M), and late (L) blocks, each containing the same number of total trials (mean  = 258; S.D.  = 28 trials). The average alpha power in each block was calculated, and each subject's binned data was normalized to his or her own “Early” block in order to normalize across individuals. Group averages were calculated, and a regression analysis was performed on the group-averaged power (12 subjects) across bins.

#### Correlation Between Alpha Power and Vigilance

In assessing the correlation between a subject's vigilance and overall alpha power, the experiment was subdivided into 100-trial bins with possible overlapping between the last and second to last bin. A regression analysis was performed between total alpha power and total blink counts in each bin across the experiment, for each subject. Total alpha power in each bin was calculated as the average across the time window [−667, 2333] ms around the cue for each trial. Blink counts were defined as the number of blinks counted in the same block of time, which in this case, includes the response periods.

#### Attention-Induced Modulation of the Time Evolution of Spectral Power

To calculate the time evolution of spectral power, percent changes from baseline (see below for definition of baseline) alpha power were calculated from −350 to 1100 ms post-cue for both of the attention cues attend-hand and attend-foot (mean  = 269 trials; S.D.  = 35 in each condition) and were averaged across bins consisting of the first 100 trials (F100), mid 100 trials (M100), and last 100 trials (L100) regardless of overlapping. In each trial bin, paired t-tests were used to calculate statistically significant differences between attend-hand vs. attend-foot conditions across subjects at every time point. Although multiple t-tests have inherent Type I errors, our data show consecutive points of significance, reducing the probability that the results represent false positives, as in our prior studies [2], [8].

#### Baseline Normalization

Two types of baseline methods were compared in our study: (1) the “all-trial baseline”, in which a common baseline is set across the two cueing conditions by averaging all trials from both conditions in order to preserve the natural starting relationship between the conditions, and (2) the “condition-specific baseline”, for which we set a baseline for each cueing condition independently in order to focus on isolating cue-induced changes in each condition. In both methods, the time window used to calculate the baseline was [−350, 0] ms in the pre-cue period.

#### Broadband A Signal of Visual Cue Evoked Responses in SI and Cue-Locked Frequency Response

Broadband signal of cue-evoked responses in SI were averaged across 100 trials for each condition in the same three bins (F100, M100, L100) as in the attention-induced modulation of the time evolution of spectral power. For each subject, each trial was baseline normalized by subtracting the mean over [−50, 0] ms in the pre-cue period. Averages across attend-hand and attend-foot conditions were calculated for each bin and averaged across subjects. Corresponding cue-locked TFR frequency responses were calculated from the averaged cue-evoked response signals for each subject, using a wavelet transform as described above. These TFRs were normalized to a “condition specific” baseline of [−650, 0] ms pre-cue, and then averaged across subjects. Paired t-tests were used to detect statistically significant differences between the two cued-attention conditions.

## Results

Preliminary analysis suggested that neocortical dynamics were more consistent across subjects at the end of an experimental session [2], [27]. Therefore, we have typically restricted our analyses of MEG data to the final 100 trials within a given session [2], [8], [21], [27]. Here, we present detailed analysis that confirms and expands upon our initial observation of these within-session dynamics.

### Within-Session Changes in Expression of Total Alpha Power

**Figure 2:**
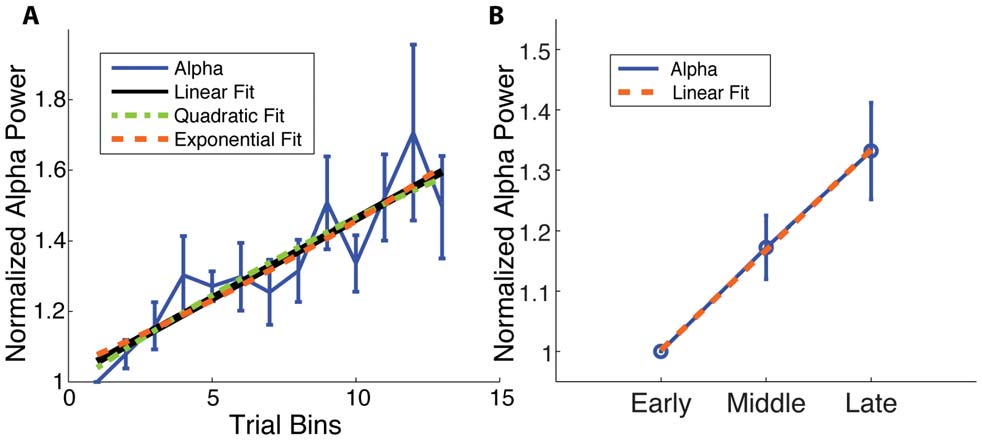
Within-Session Increase in Total Alpha Power. **A**) Within-Session Increase in Binned Total Alpha Power. The group average of binned alpha power is fitted to a linear (black), quadratic (green), and exponential (orange) model. Blue Trace: Average alpha power (7–14 Hz, mean and standard error (S.E.), n = 12 subjects) from the hand area in SI across the experiment that has been divided into bins of 50 trials. For each subject, alpha power was normalized to the earliest bin average. No significant improvement beyond linear model is seen. (Linear fit: R^2^ = 0.81, p = 0.03, one-sided t-test) **B**) Within-Session Increase in Expression of Total Alpha Power. Blue Trace: Average alpha power (7–14 Hz, mean and S.E., n = 12 subjects) from the hand area in SI across three blocks of the experiment (early (E), middle (M), and late (L) trials). For each subject, alpha power was normalized to the early block (E) average. Linear regression analysis (orange dash) confirmed a statistically significant increase in total alpha power across the experimental session (R^2^ = 0.99, p<0.01, one-sided t-test).

To assess the changes in alpha power across an experimental session, we first investigated changes in total alpha power throughout the entire session for each subject during the cued-attention task. For each trial, we calculated the average power in time period of [−667, 2333] ms around cue across all conditions and binned the data by averaging every 50 trials. In 11 out of 12 subjects (p = 0.003, sign test) we observed an increased power from the beginning to the end of the experiment. To quantify the evolution of alpha power over time, the group average of the normalized alpha bins were fit to a linear, a quadratic, and an exponential model. The nonlinear models produced fits similar to that of a linear model. (Figure 2A: Linear regression analysis (black line) R^2^ = 0.81, p = 0.03, one-sided t-test).

To be consistent with our other examinations of changes in alpha dynamics across the experiment, we also divided the entire experimental session into three blocks of equal numbers of trials: early (E), middle (M), and late (L) (mean  = 258 per block; S.D.  = 28), and calculated grand average alpha power in each block. We again found that the mean alpha power increased linearly across the experimental session (Figure 2B: Linear regression analysis (orange dash) R^2^ = 0.99, p = 0.03, one-sided t-test).

A common view of alpha oscillations is that they are an index of arousal, therefore increased alpha power within a session could reflect increased drowsiness [31]. To check whether the observed increase in SI alpha power was linked to decreased vigilance, we performed a correlation analysis between blink count and alpha power [32], using eye blink as an indicator for drowsiness, as described in the Methods. We found no correlation between alpha power increase and blink rate across a session for 11 of 12 subjects (p>0.05; data not shown).

### Within-Session Changes in the Differential Allocation of Alpha Power Following Directed Attention Cueing

Next, we studied within-session changes in cue-induced alpha modulation as observed in Jones et al. (2010) [2]. In our prior study, we found that SI alpha power was significantly higher in the somatotopic representation of the hand when attention was cued to the body position of the foot than when attention was cued to the hand. This differentiation in post-cue alpha modulation began ∼600 ms post-cue, during the anticipatory period prior to a tactile stimulus. In that study, we reported on only the last 100 trials for each subject, following the observation made on a related prior study regarding data stability [27].

**Figure 3:**
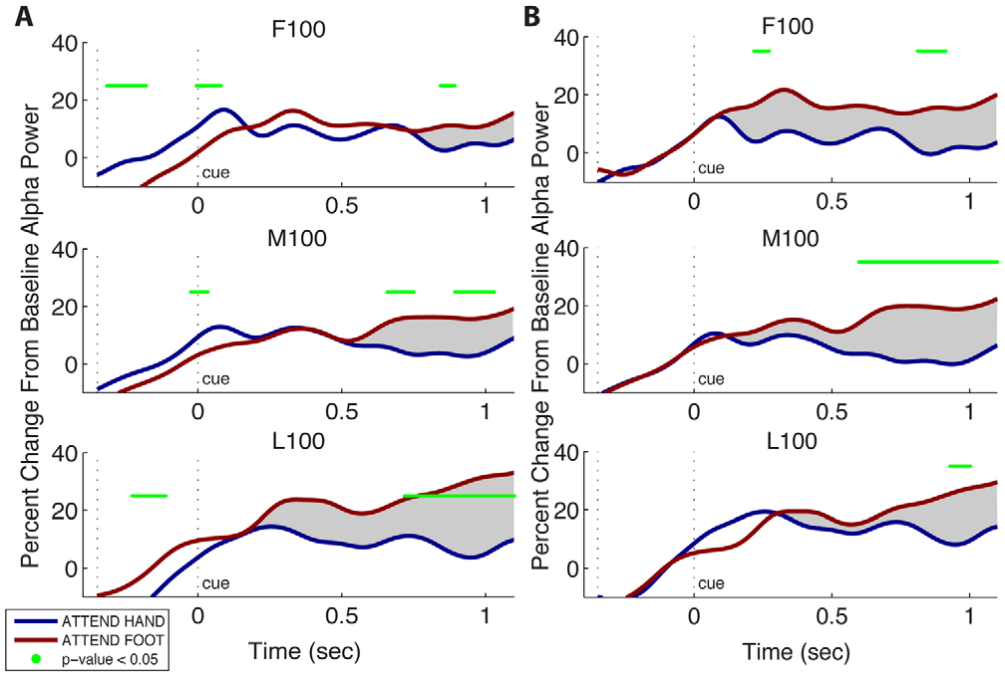
Within-Session Changes in Cue-Induced Allocation of Alpha Power and Implication of Different Baseline Methods. **A**) Within-session changes in differential allocation of alpha power following directed attention cueing using “all-trial baseline” method. Post-cue temporal evolution of the hand area SI alpha activity in attend-hand and attend-foot conditions in three blocks of the experiment: First 100 trials (F100), mid 100 trials (M100), and last 100 trials (L100). Alpha power was plotted as a percent change from baseline using an “all-trial baseline” (average across all conditions, see Methods). Green Asterisks: Significant differences between attend-hand and attend-foot conditions (p<0.05, paired t-test). Significant differences evolved across the duration of the experiment with the longest time period of significance in the L100 trials. **B**) Within-session changes in differential allocation of alpha power following directed attention cueing using “condition-specific baseline” method. Different baseline methods showed different within-session progression of the dynamic allocation of alpha with cued attention. Green Asterisks: Significant differences between attend-hand and attend-foot conditions (p<0.05, paired t-test). The trend in differentiation of alpha modulation post-cue is highlighted by the gray shading.

Here, we performed the same analysis as in Jones et al. (2010) [2] on data that were divided into three time blocks, with each block containing 100 trials (first (F100), middle (M100), and last (L100) trials). Our L100 results (Figure 3A bottom panel) replicated the results in Jones et al. (2010), albeit with an updated artifact region method (see Methods). The lower number of trials included in each block in this analysis compared to the total power analysis (mean 258 trials) reflects the decrease in overall N due to division of trials into attend-hand and attend-foot conditions — a division that was not necessary when examining overall alpha power in Figure 2.

When separated into three time blocks, we found that the differential alpha allocation with attention that we observed in the L100 trials was not consistent in all three stages of the experiment. Although the ability to modulate SI alpha was present in all three time blocks, there was a systematic evolution in the timing of significant differences between attention conditions (Figure 3A). This evolution occurred in time periods around the cue, including pre-cue, and in later >500 ms post-cue activity.

In the later >500 ms post-cue activity, the attention-induced differential alpha allocation occurred earlier and over a longer time window as the experiment progressed. In F100, the significance occurred very briefly between [840, 890] ms post-cue. In M100, the significance occurred earlier at [650, 1030] ms and was more consistently in time, although still had brief interruptions. By L100, the separation was sustained the entire time from approximately 750 ms to the end at 1100 ms, and an even earlier point of differentiation was visible although not reaching statistical significance. The growing trend in duration and magnitude of the differentiation in alpha allocation to cued attention is highlighted by the gray shaded region in Figure 3A.

In the time period at and before the cue, the differential alpha allocation with attention flipped sign from the beginning to the end of the experiment, such that in the F100 the attend-hand condition was higher than attend-foot condition, and by the L100 the attend-foot condition was higher than attend-hand. In the F100 statistically significant differences occurred between approximately [−320, −180] ms pre-cue and briefly at [−5, 80] ms around cue. In M100, while the attend-hand condition was still greater, the difference became less apparent with no significant difference in pre-cue period and an even briefer period of significance around the cue from approximately [−20, 20] ms. By L100 trials the difference flipped sign and was again significant in the pre-cue period where the attend-foot condition was greater than the attend-hand condition from approximately [−200, −110] ms.

**Figure 4:**
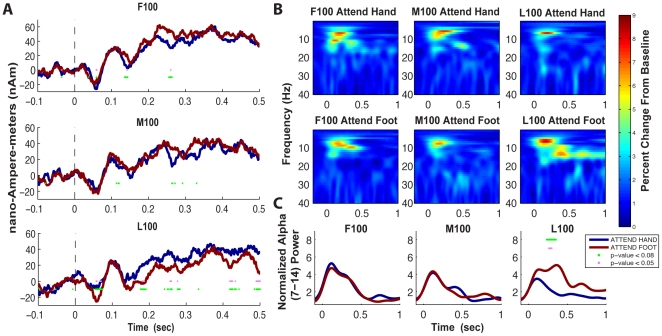
Within-Session Changes in Cue-Evoked Broadband Signal and Corresponding Frequency Response. **A**) Average of hand area SI broadband evoked response from the visual cue in attend-hand and attend-foot conditions (mean n = 12 subjects) in the three blocks (F100, M100 and L100 trials) of the experiment. **B**) Corresponding normalized TFR spectrogram of the immediate cue-locked evoked response (calculated individually for each subject then averaged; n = 12 subjects). **C**) Normalized alpha power (mean 7–14 Hz) for the attend-hand and attend-foot conditions. Pink Asterisks: Significant differences between attend-hand and attend-foot conditions (p<0.05, paired t-test). Green Asterisks: Trend differences (p<0.08). Differences in immediate cue-locked alpha power evolved across the duration of the experiment emerging significantly only in the L100 trials.

We note that since the experimental design randomized trial presentation order and timing, the significant differences in the pre-cue baseline period and immediately surrounding the cue did not reflect the subjects' ability to predict the upcoming trial. Rather, these differences can be explained by difference in alpha power time locked to the cue that smeared into the baseline period because of limitations in the temporal resolution of the wavelet methods used. For example, since we used a 7 cycle wavelet, to estimate 10 Hz alpha power at any point in time, a 700 ms time window of data is necessary. Therefore, at the cue, the estimation of alpha was smeared up to 350 ms pre- and post-cue, encompassing the length of our baseline period and the difference in the pre-cue baseline results from differences that actually occurred at and immediately after the cue. This fact can be visualized in the broadband cue evoked responses in Figure 4A. In each panel of Figure 4A, a cue locked oscillation with a period of ∼100 ms immediately following the cue was visible for both attend-hand and attend-foot conditions. The amplitude of these cue induced oscillations changed across the experiment such that in the F100 trials, the amplitude of the attend-hand oscillation was larger, and by the L100 trials the amplitude of the attend-foot conditions was larger. This transformation was reflected in the frequency analysis in Figure 3A. The frequency analysis was calculated on each trial separately and thus reveals effects that may or may not be time-locked to the cue. In this case, we see that at least part of the significant differences near the cue in Figure 3A is due to differences in evoked oscillations time locked to the cue.

### Baseline Normalization

During our exploration of the within-session post-cue attention modulation of alpha power, we also observed that the choice of baseline normalization could cause distinct differences in the results, particularly in the time period around the cue. One of two types of normalization methods are typically employed when comparing two conditions: (1) An “all trials” baseline, where a single baseline calculated from averages of all conditions is used for normalizing data [2], as in Figure 3A or (2) a “condition specific” baseline, where specific conditions are normalized to their own baseline pinning each condition to a common zero starting point [5], [22], as in Figure 3B.

We observed a difference in the progression of within-session dynamics using these two different baseline methods. While attention induced modulation of alpha was present throughout the experiment using both normalization methods, in contrast to the “all trials” baseline, where an evolution of differences in both the early (pre-cue and around the cue) and the late (>500 ms post-cue) time windows were apparent (Figure 3A), the “condition specific” baseline emphasized differences in the later post-cue activity only (Figure 3B).

Due to the fact that the “condition specific” baseline method forces the data from each condition to start at a common zero point, differences at the time of the cue were no longer visible. However, the later post-cue differences between attention conditions, which occurred >500 ms in Figure 3A, are now significant at earlier time points beginning as early as 210 ms in the first block of the experiment (F100). The starting point of significant differences moves to later time points as the experiment processes, beginning at 600 ms during M100 and at approximately 930 ms by the L100 in Figure 3B. The fact that the duration and magnitude of pos-cue differentiation between conditions appeared to shrink from the F100 to L100 trials is the complete opposite of what was observed in the “all-trial” baseline methods. The contrast is highlighted by the comparing the gray shading seen in Figure 3A and 3B.

These results showed that while the attention-induced allocation of alpha was present in both baseline conditions across the experiment, when studying the dynamics of the evolution of this phenomena across the experimental session, the baseline condition chosen can lead to different conclusions.

### Within-Session Changes in Visual-Cue-Evoked Broadband Signal and Corresponding Frequency Responses in SI

In addition to attention-induced alpha modulation, we also previously reported significant differences in the immediate cue-evoked SI hand responses between attend-hand and attend-foot conditions [2]. Here, we investigated if this significant difference also evolved over the course of the experiment.

We found that, as described in Jones et al. (2010) [2], there were significant differences in several peak values between attend-hand and attend-foot conditions when comparing the averaged broadband signal of cue-evoked responses in the L100 trials. During L100 trials, the attend-foot waveform was lower than the attend-hand waveform, and the magnitude of peak activity was significantly larger in the attend-foot condition at ∼70 ms, ∼200 ms, ∼250 ms, ∼400 ms, and ∼500 ms activity (Figure 4A bottom panel), as described in our prior report. When comparing these differences in the cue-evoked response to the F100 and M100 trials, we again found that there was an evolution of differences across the experiment. In the F100 trials, the cue-evoked waveforms were closely aligned during the first 200 ms, and the attend-foot condition emerged higher at ∼250 ms (Figure 4A, top panel). During the M100 trials, the attend-foot waveform appeared to become more oscillatory particularly from ∼250–350 ms, where larger windows of difference emerged again with the attend-foot condition greater than attend-hand conditions (Figure 4A, middle panel). By the L100 trials, earlier differences emerged, beginning at ∼70 ms, and now the early oscillation in the attend-foot condition was clearly larger in magnitude and the entire waveform was consistently lower than the attend-hand condition showing a reversal from the F100 and M100 trials.

Further, as described above in the discussion of Figure 3A, visual inspection of the broadband cue-evoked responses in Figure 4A showed the emergence of an immediate cue-locked oscillation with a period of ∼100 ms, placing it in the alpha-band. To confirm the presence of this cue-locked oscillation, and to investigate its evolution across the experiment, we calculated a time-frequency response spectrogram (TFR) on the averaged broadband signals for each subject (unlike Figure 3 where TFRs were calculated separately for each trial, see Methods), normalized each subject by converting the TFRs to percent change from the “condition specific” baseline. The spectrogram of the group average of this cue-locked activity across subjects is shown in 4B. Responses averaged over only the alpha band are in depicted in Figure 4C. In the alpha band, significant differences across conditions emerged only in the L100 trials (p<0.05) in a small time window around 275 ms with a longer trend period (p<0.08) (Figure 4C, panel 3).

## Discussion

Human imaging studies that use EEG, MEG, and fMRI often average event-related activity across trials taken from an entire experimental session. Here, we demonstrate a key potential shortcoming of this approach. We found within-session variation in many aspects of the SI alpha rhythm, including total power, the degree of differentiation in attention-induced alpha allocation, the peak differences in broadband signals of visual cue-evoked response, and cue-locked frequency responses in the alpha band. Further, we found that the evolution of alpha dynamics across the experiment were distinctly different when using two conventional baseline normalization methods — a phenomenon that can introduce different interpretations regarding short-term learning and adaptation. These findings are not only relevant for understanding this commonly measured rhythm, but is, more generally, a case study in the necessity of tracking within-session analyses in human scanning. It is a kind of analysis that is almost never carried out, and yet could drastically impact data interpretation and the choice of analysis technique.

### Relevance of Within Session Changes in Total Alpha Power and Attention-Induced Modulation of Alpha

Two existing hypotheses as to the functional relevance of alpha oscillations could explain the increased alpha power observed across an experimental session. Classic theory treats alpha power as an indicator for states of drowsiness [1] and active disengagement from the task [33]. Our analysis between SI alpha power and blink count did not show any evidence for a correlation between vigilance measured by blink count and alpha power, and the absence of a behavioral indicator of decreased vigilance and increased drowsiness suggested that the systematic increase of alpha across the session was not a generalized decline of arousal state. However, our threshold stimuli were actively manipulated to maintain a 66% detection rate, so the data we collected did not include enough psychophysical or behavioral information to either support or refute an influence of alpha rhythm activity due to active disengagement from the task on performance.

The second theory comes from recent studies that have demonstrated alpha modulation induced by attention demand, which can be restricted to specific neocortical regions. Results from these studies suggested that an increase in alpha in a specific region could be correlated with shifting attention away from that representation, presumably to enhance signal-to-noise-ratio through the suppression of distracting stimuli [2], [3], [5], [6].

Our data, while unable to fully address the question of underlying mechanisms, favored the second view. Our finding of a steady increase in total alpha power suggested more effort was exerted to suppress distracting stimuli, and that the subjects' were progressively more effective in recruiting this rhythm through learning to control brain dynamics within a session. Further, we found that the subjects' ability to modulate alpha according to cue accompanied the increased overall alpha power, which suggested that the increased alpha power measured through the session was explicitly linked to changes in the ability to allocate alpha with an attention cue. This evolution in dynamics with attention allocation may imply a rapid form of perceptual learning and adaptation even in non-training paradigms.

### Relevance of Baseline Normalization

By comparing two common baseline methods, we found that while both methods showed alpha allocation with attention throughout the experiment, conclusions on the evolution of these the dynamics across the session were baseline dependent. The “all trial” baseline preserved the natural relationship between the two conditions at all times and showed significant differentiation before, during, and after the onset of the cue. The evolution of these high differentiation periods suggested that the attention-induced allocation of alpha improved progressively through the entire ∼1 h session of the experiment, increasing in duration and magnitude. Further, the immediate cue-evoked response in alpha, which smeared into the pre-cue period, switched from being higher in attend-hand condition to higher in attend-foot conditions (Figure 3A), reflective of changes in the immediate cue-locked evoked response (Figure 4A). The “condition specific” baseline method focused on isolating the dynamics occurring post-cue because it pinches the conditions to a common zero point at the cue (Figure 3B). This method suggested that there is a mechanism that produces a rapid cue-induced alpha allocation occurring around 200 ms post-cue that only occurred in the beginning of the experiment and disappeared as the experiment progress. With this method, the duration and magnitude of the significance differences across attention conditions appeared to progressively decrease across the experiment.

Our results suggested that studies that examine the temporal evolution of neocortical dynamics should carefully consider the baseline procedures used, as different interpretation on adaptation and short-term learning can be inferred depending on the baseline methods used.

### Relevance of Within Session Changes in Visual-Cue-locked Broadband Evoked Response

Rapid responses (<100 ms) to visual stimuli in SI have been observed previously in primates, and have been correlated to performance in tactile perception tasks [34], [35]. The increased response to different visual cues in SI in our data, in the broadband signal and frequency domain, suggested that within-session learning effects influence the emergence of these rapid cross-modal responses. The observed immediate increase in cue-locked alpha oscillations in the SI hand representation during the visual cue to attend-foot was consistent with our view that alpha was being specifically allocated to unattended regions to decrease distraction in those regions. Our results suggested that this more rapid response is a dynamic process that only becomes statistically significant after training. This result suggested that there is a potentially different mechanism that induces a cue-locked alpha response in SI following a visual cue, which occurs in addition to the mechanism that produces a non-time-locked attention-induced alpha modulation at a later onset.

### Implications of these Findings for Studies of Neural Dynamics

Shifts in the expression of neural oscillatory dynamics on the time scale of 1–2 hours are well documented in studies of learning and vigilance [23], [26], however these changes are seldom explicitly factored into the design of studies that involve scanning sessions lasting this duration, therefore interesting dynamics may be overlooked.

While the data presented here do not change the overall conclusion of our prior study showing allocation of alpha with attention, they make explicit suggestions that can reveal important shorter time scale evolving dynamics that are canceled when averaging over the entire session. First, our results indicated that within session time-dependence of dynamics should be considered prior to assuming that event-related averaging is proper, as interesting differences may be evident in only a specific segment of the experiment and would be otherwise overlooked. This implication applies not only studies conducted using MEG or EEG, but also to the myriad fMRI studies published using similar data culling methods. Second, our data suggested that even paradigms without an overt or intended learning component might demonstrate an evolution in the allocation of dynamics that appears to reflect an explicit or implicit learning process. Third, our data suggested that relatively subtle differences in normalization techniques, which are generally essential when averaging data across sessions or subjects, can lead to significant differences in the interpretation of oscillatory dynamics and in the progression of their within-session changes. As such, these findings recommend the explicit comparison of these and related normalization techniques as part of a data analysis approach to studying the evolution of dynamics.
